# Pet ownership, loneliness, and social isolation: a systematic review

**DOI:** 10.1007/s00127-022-02332-9

**Published:** 2022-07-11

**Authors:** Benedikt Kretzler, Hans-Helmut König, André Hajek

**Affiliations:** Department of Health Economics and Health Services Research, University Medical Center Hamburg-Eppendorf, Hamburg, Germany

**Keywords:** Pet ownership, Animal ownership, Loneliness, Social isolation, Social exclusion, Systematic review

## Abstract

**Purpose:**

Several publications explored a relationship between pet ownership and lower levels of loneliness and social isolation. However, to the best of our knowledge, no systematic review has yet synthesized the evidence on these associations. Thus, this systematic review aims to evaluate the findings regarding the relations between pet ownership, loneliness, and social isolation.

**Methods:**

PubMed, CINAHL, and PsycInfo were searched in January 2022. Observational studies relying on appropriate instruments to assess the exposure and the outcome variables were included. Two reviewers independently executed study selection, data extraction, and quality assessment.

**Results:**

*n* = 24 studies were included. Among adult samples, the studies examining the relationship between pet ownership and social isolation found that owning a pet was associated with lower levels of social isolation. Concerning loneliness, studies that were conducted after the outbreak of COVID-19 mostly showed that pet ownership can contribute to lower levels of loneliness, but did not reveal an overall significant association until then. In turn, the studies that examined child and adolescent samples suggest that pet ownership was related to reduced loneliness before COVID-19. Furthermore, most of the studies did not reveal any differences between dogs, cats, and other kinds of pets regarding their relationship to loneliness and social isolation.

**Conclusion:**

All in all, only a part of the studies detected a significant association between pet ownership, loneliness and social isolation. However, the COVID-19 pandemic seemed to strengthen this relationship, so that future research is required to assess the longevity of this potential effect.

## Introduction

The Oxford English Dictionary defines a pet as “an animal (typically one which is domestic or tame) kept for pleasure or companionship” and hereby separates it from animals that one gets in touch with among natural or professional environments [1]. Pets are widely spread in several countries, such as the United States: According to the American Veterinary Medical Association, two-thirds of the American households owned a pet in 2018, with nearly two out of five households possessing one or more dogs, and one quarter of the households at least one cat [2]. After the outbreak of COVID-19 in 2020, the share of American households owning a pet climbed to an all-time high of 70% [3].

Research showed that possessing a companion animal can lead to various positive health outcomes—a phenomenon that has already been called the “pet effect” previously [4]. This pet effect concerns physical, psychological and social health [5]: For instance, in 2018 and 2019, two studies were published that revealed an association between pet ownership and lower levels of frailty [6] and higher levels of physical activity [7]. In addition, pets may also enhance mental health components. Pet ownership was shown to be related to lower levels of depressive symptoms [7] and anxiety [8]. Eventually, an investigation carried out in Australia found pet owners to be more likely to get in touch with people living in their neighborhood [9]. Besides these general associations, the value of pets seems to further increase when their owner goes through straining times: for example, Siegel showed that individuals suffering from stress reported fewer physician visits when they owned a companion animal [10], and another study even revealed that pet ownership was associated with higher likelihoods of survival after cardiovascular events [11].

A widely used theoretical framework to explain such beneficial effects of animal companionship on humans is the so-called Attachment Theory, which assumes that humans have a need of being attached or belonging to someone [12]. Regardless of the obvious differences between human-to-human and human–animal interactions, pets may partly satisfy these needs as well, providing some kind of social support that was shown to be related to physical and mental health variables [13]. There are even studies which suggest that individuals with high attachment to pets do not perceive large differences between human-to-human and human–animal interactions [14]. The association between pet ownership and social support may be particularly important among older people who did not marry, are divorced or became widowed, as they tend to show higher levels of attachment towards pets and have a higher probability of anthropomorphizing them [15], which could be related to previous findings that pets can buffer the negative effects of missing social support: For instance, Bryan et al. revealed that individuals with high levels of ambivalence over emotional expression (AEE), who tend to suffer from a lack of social support, can receive exactly this kind of support by their pets, especially in case of a high affinity to them [16]. Regarding lack of social support and pet affinity as general topics that do not only occur among individuals with AEE, this effect seems to be applicable to other populations as well.

Conversely to the larger part of research works that focused on these health-related outcomes, several articles also covered the association between pet ownership, loneliness, and social isolation, e.g., [7, 17–21]. What is more, the number of such publications seems to have increased since the beginning of the COVID-19 pandemic, as many studies examining this relationship have been released since that, e.g., [22–24]. This development may not appear very surprising, as the importance of loneliness in the public discourse strongly rose during a time of lockdowns and “stay-at-home” policies [25, 26]. Nonetheless, there is mixed evidence regarding an increase in loneliness since March 2020, with some studies reporting high levels of loneliness particularly during the initial phases of lockdown [27] and some others detecting strong resilience among the population in response to social distancing [28]. Notwithstanding their interchangeable use in a part of the literature [29] and their association with social needs in general [30], loneliness and social isolation define differing concepts. On the one hand, loneliness bears on a subjective perception that the quality of one’s social relationships is not sufficient [31]. Consequently, this means that feeling lonely is not automatically precluded by a high social network size. However, there is also a quantitative aspect, as a small social network may be linked to an unsatisfactory quality of social relationships. [32]. On the other hand, social isolation refers to the feeling of not belonging to society, especially through a low number of social interactions and a limited social network size [33]. Though, it can be further differentiated into objective social isolation, which mainly refers to indicators as those listed in the previous sentence [34], and into perceived social isolation, which means the feeling of not belonging to the society [35]. All in all, the concepts of loneliness and social isolation are related as they share common aspects such as suggesting smaller network sizes, but it is social isolation that explicitly focuses on these quantitative aspects, while loneliness should better be seen as a perception of a deficient social inclusion that does not fulfill the standard desired by an individual.

Regarding previous studies of the relationship between pet ownership and these concepts, there are different findings. On the one hand, some studies explored a relief of loneliness [36, 37] or social isolation [7, 21] through pet ownership. On the other hand, there are also several studies that did not detect a significant impact of pets on the same constructs [17, 38], and one paper even revealed worse outcomes regarding loneliness among those having a pet [39]. Despite the number of papers that investigated that topic and the mixed evidence that they obtained, there is no systematic review which systematically summarizes the findings to assist in filling the knowledge gap, which still exists concerning the association between pet ownership and loneliness as well as social isolation. Given that, the aim of our article is to provide that kind of systematic review, synthesizing the findings of observational studies. This could help in identifying individuals at risk of not being able to satisfy their social needs. In addition, it could inspire and guide future research in this area.

## Materials and methods

Our review meets the standards of the Preferred Reporting Item for Systematic Reviews and Meta-Analysis Protocols guidelines [40] and is registered with the International Prospective Register of Systematic Reviews (PROSPERO, registration number: CRD42020193102).

### Search strategy and selection criteria

Three databases (CINAHL, PsycInfo, and PubMed) were employed to find relevant literature. The search was executed in January 2022. Our search algorithm is reported in Table 1.

**Table 1:** Search algorithm (PubMed)

#1	Loneliness
#2	Social isolation
#3	Social exclusion
#4	Social frailty
#5	#1 OR #2 OR #3 OR #4
#6	Pet ownership
#7	Dog
#8	Cat
#9	“Animal owner*”
#10	#6 OR #7 OR #8 OR #9
#11	#5 AND #10

The titles and abstracts of the articles that were delivered through the databases were screened with respect to the inclusion and exclusion criteria, which are described in the subsequent paragraphs. After that, the full texts of the articles that had passed the title-abstract screening were considered to define the final sample of articles regarding the association between pet ownership, loneliness, and social isolation. The title-abstract screening as well as the full-text screening were performed independently by two reviewers (BK and AH). Beyond that, the reference lists of the studies in the final sample were considered as well. Any disagreements between the two reviewers that occurred during both screening processes could be resolved through discussion.

As for the inclusion criteria, our review covers observational studies, cross-sectional as well as longitudinal, which describe the relationship between pet ownership, loneliness, and social isolation.

We excluded:

- studies not describing this relationship
- studies focusing on illness-specific samples, as the aim of our review is to synthesize the evidence on the general relationship between pet ownership, loneliness, and social isolation, and not to study how it turns out among particular subgroups, e.g., individuals with a particular disease such as dementia
- studies not having an observational design
- studies not published in German or English
- studies not published in scientific, peer-reviewed journals

Yet, concerning the time or the location of a study (reported in article), no restrictions we applied.

The inclusion and exclusion criteria were pretested by employing them for the first 100 articles of the title-abstract screening. As there were no major differences between the two reviewers (BK and AH), we abstained from any change afterwards.

### Data extraction and analysis

The data extraction was carried out by one reviewer (BK) and cross-checked by a second one (AH). We extracted data about a study’s time of publication, region, assessment of predictor and outcome variables, design, sample, and key results on the association between pet ownership, loneliness, and social isolation.

### Quality assessment

To assess the studies’ quality, we used the well-established and widely used NIH Quality Assessment Tool for Observational Cohort and Cross-Sectional Studies [41]. The quality assessment was performed independently by two reviewers (BK and AH).

## Results

The following subsections will provide the results stratified by adult (mean age varied between 25.1 and 76.6) and youth samples (mean age varied between 12.7 and 21.3).

### Included studies

**Fig. 1:**
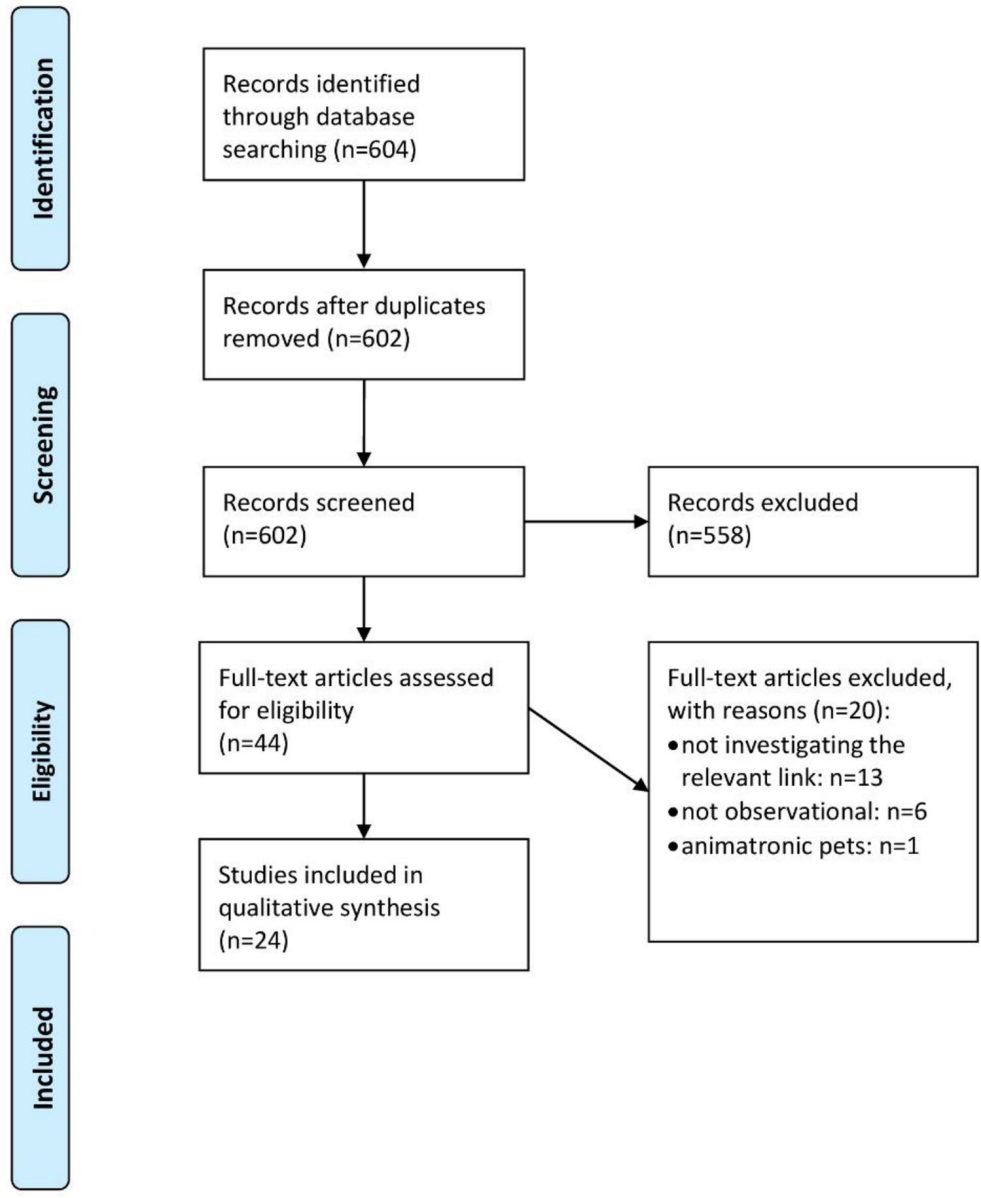
PRISMA flow diagram

The course of the screening process is provided in Fig. 1 (PRISMA 2009 Flow Diagram) [42]. Searching CINAHL, PsycInfo, and PubMed provided *n* = 604 hits. After the title-abstract screening, *n* = 44 of them were remaining, while *n* = 558 articles were excluded, mainly because neither their titles nor their abstracts mentioned an investigation concerning the relationship between pet ownership and loneliness or social isolation, or because the information provided in title or abstract pointed towards hitting the exclusion criteria, especially with respect to the requirement of an observational design. During the full-text screening, *n* = 13 studies were excluded because they did not contain results concerning the relationship between pet ownership, loneliness, and social isolation. Furthermore, *n* = 1 article only described the association between having an animatronic pet and the two outcome variables. Eventually, *n* = 6 studies were not observational and did, therefore, not meet the inclusion criteria as well. However, no study had to be excluded because it was not using appropriate tools to quantify the variables of interest, only investigating a specific sample, or published in a non-scientific journal or neither in English nor in German language. Our final sample consisted of *n* = 24 studies.

### Quality assessment

The quality assessment of the studies included in the final sample is displayed in Table 2. Half of the criteria (e.g., clearly specifying and defining the study population or having clearly defined, valid, reliable, and consistently implemented exposure and outcome measures) were fulfilled by (almost) every article included. Notwithstanding this, some other criteria were hardly met by any study (e.g., sufficient timeframe, exposure(s) assessed more than once over time). All in all, the study quality was satisfactory: one-half of the studies [7, 17, 21, 22, 24, 39, 43–48] was rated as “good”, the other half [18–20, 23, 36–38, 49–53] as “fair”.

**Table 2:** Quality assessment

Paper author and date	(1) Was the research question or objective in this paper clearly stated?	(2) Was the study population clearly specified and defined?	(3) Was the participation rate of eligible persons at least 50%?	(4) Were all the subjects selected or recruited from the same or similar populations (including the same time period)? Were inclusion and exclusion criteria for being in the study prespecified and applied uniformly to all participants?	(5) Was a sample size justification, power description, or variance and effect estimates provided?	(6) For the analyses in this paper, were the exposure(s) of interest measured prior to the outcome(s) being measured? (If not prospective should be answered as ‘no’, even is exposure predated outcome)	(7) Was the timeframe sufficient so that one could reasonably expect to see an association between exposure and outcome if it existed?
Antonacopoulos (2010) [50]	Yes	Yes	Not reported	Yes	No	No (cross-sectional)	No (cross-sectional)
Antonacopoulos (2017) [49]	Yes	Yes	Not reported	Yes	No	Yes	Yes
Bennett (2015) [38]	Yes	Yes	Not reported	Yes	No	No (cross-sectional)	No (cross-sectional)
Black (2012) [36]	Yes	Yes	Yes	Yes	Yes	No (cross-sectional)	No (cross-sectional)
Branson (2019) [43]	Yes	Yes	Not reported	Yes	Yes	No (cross-sectional)	No (cross-sectional)
Carr (2021) [22]	Yes	Yes	Not reported	Yes	No	No (simultaneously)	Yes
Carr (2020) [44]	Yes	Yes	Not reported	Yes	No	No (simultaneously)	Yes
Charmaraman (2020) [51]	Yes	Yes	Not reported	Yes	No	No (cross-sectional)	No (cross-sectional)
Enmarker (2015) [19]	Yes	Yes	Yes	Yes	No	No (cross-sectional)	No (cross-sectional)
Gulick (2012) [52]	Yes	Yes	Not reported	Yes	No	No (cross-sectional)	No (cross-sectional)
Hajek (2020) [21]	Yes	Yes	Yes	Yes	No	No (cross-sectional)	No (cross-sectional)
Kogan (2021) [23]	Yes	Yes	Not reported	Yes	No	No (cross-sectional)	No (cross-sectional)
McConnell (2011) [53]	Yes	Yes	Not reported	Yes	No	No (cross-sectional)	No (cross-sectional)
Mueller (2021) [45]	Yes	Yes	Not reported	Yes	No	Yes	Yes
Oliva (2021) [46]	Yes	Yes	Not reported	Yes	No	No (cross-sectional)	No (cross-sectional)
Phillipou (2021) [24]	Yes	Yes	Not reported	Yes	No	No (cross-sectional)	No (cross-sectional)
Pikhartova (2014) [39]	Yes	Yes	Not reported	Yes	No	No (simultaneously)	Yes
Powell (2018) [20]	Yes	Yes	Not reported	Yes	No	No (cross-sectional)	No (cross-sectional)
Ratschen (2020) [47]	Yes	Yes	Not reported	Yes	No	No (cross-sectional)	No (cross-sectional)
Rhoades (2015) [37]	Yes	Yes	Yes	Yes	No	No (cross-sectional)	No (cross-sectional)
Rijken (2011) [48]	Yes	Yes	Yes	Yes	No	No (cross-sectional)	No (cross-sectional)
Stanley (2014) [18]	Yes	Yes	Not reported	Yes	No	No (cross-sectional)	No (cross-sectional)
Taniguchi (2018) [7]	Yes	Yes	Yes	Yes	No	No (cross-sectional)	No (cross-sectional)
Zasloff (1994) [17]	Yes	Yes	Yes	Yes	No	No (cross-sectional)	No (cross-sectional)

Paper author and date	(8) For exposures that can vary in amount or level, did the study examine different levels of the exposure as related to the outcome (e.g., categories of exposure, or exposure measured as continuous variable)?	(9) Were the exposure measures (independent variables) clearly defined, valid, reliable, and implemented consistently across all study participants?	(10) Was the exposure(s) assessed more than once over time?	(11) Were the outcome measures (dependent variables) clearly defined, valid, reliable, and implemented consistently across all study participants?	(12) Was loss to follow-up after baseline 20% or less?	(13) Were key potential confounding variables measured and adjusted statistically for their impact on the relationship between exposure(s) and outcome(s)?	Overall quality judgment
Antonacopoulos (2010) [50]	Continuous	Yes	No	Yes	Not applicable (cross-sectional)	Yes	Fair
Antonacopoulos (2017) [49]	Continuous	Yes	Yes	Yes	No	No	Fair
Bennett (2015) [38]	Continuous	Yes	No	Yes	Not applicable (cross-sectional)	No	Fair
Black (2012) [36]	Continuous	Yes	No	Yes	Not applicable (cross-sectional)	No	Fair
Branson (2019) [43]	Continuous	Yes	No	Yes	Not applicable (cross-sectional)	Yes	Good
Carr (2021) [22]	Continuous	Yes	Yes	Yes	No	Yes	Good
Carr (2020) [44]	Continuous	Yes	Yes	Yes	No	Yes	Good
Charmaraman (2020) [51]	Continuous	Yes	No	Yes	Not applicable (cross-sectional)	Yes	Fair
Enmarker (2015) [19]	Dichotomous	Yes	No	Yes	Not applicable (cross-sectional)	No	Fair
Gulick (2012) [52]	Continuous	Yes	No	Yes	Not applicable (cross-sectional)	No	Fair
Hajek (2020) [21]	Continuous	Yes	No	Yes	Not applicable (cross-sectional)	Yes	Good
Kogan (2021) [23]	Dichotomous	Yes	No	Yes	Not applicable (cross-sectional)	Yes	Fair
McConnell (2011) [53]	Continuous	Yes	No	Yes	Not applicable (cross-sectional)	No	Fair
Mueller (2021) [45]	Continuous	Yes	Yes	Yes	No	Yes	Good
Oliva (2021) [46]	Continuous	Yes	No	Yes	Not applicable (cross-sectional)	Yes	Good
Phillipou (2021) [24]	Continuous	Yes	No	Yes	Not applicable (cross-sectional)	Yes	Good
Pikhartova (2014) [39]	Dichotomous	Yes	Yes	Yes	Not reported	Yes	Good
Powell (2018) [20]	Dichotomous	Yes	No	Yes	Not applicable (cross-sectional)	Yes	Fair
Ratschen (2020) [47]	Continuous	Yes	No	Yes	Not applicable (cross-sectional)	Yes	Good
Rhoades (2015) [37]	Continuous	Yes	No	Yes	Not applicable (cross-sectional)	No	Fair
Rijken (2011) [48]	Continuous	Yes	No	Yes	Not applicable (cross-sectional)	No	Good
Stanley (2014) [18]	Dichotomous	Yes	No	Yes	Not applicable (cross-sectional)	Yes	Fair
Taniguchi (2018) [7]	Dichotomous	Yes	No	Yes	Not applicable (cross-sectional)	Yes	Good
Zasloff (1994) [17]	Continuous	Yes	No	Yes	Not applicable (cross-sectional)	No	Good

### Adult population

#### Adult population prior to the pandemic

Overall, *n* = 15 of the studies included in our final sample provided results on the association between pet ownership, loneliness, and social isolation among an adult population prior to the pandemic [7, 17–21, 38, 39, 43, 44, 48–50, 52, 53].

Data were derived from the United States (*n* = 6) [17, 18, 43, 44, 52, 53], Canada (*n* = 2) [49, 50], Australia (*n* = 2) [38, 46], the United Kingdom (*n* = 1) [39], Germany (*n* = 1) [21], Japan (*n* = 1) [7], the Netherlands (*n* = 1) [48], and Norway (*n* = 1) [19].

With *n* = 2 exceptions [43, 49], all studies provided results about the relationship between owning any kind of pet and loneliness or social isolation. Out of these *n* = 14 studies, only one did not just relate to owning a pet at the timepoint when the study was conducted, but to pet ownership in the past [7]. Besides that, *n* = 1 study investigated the interaction between pet ownership and a social loss [44]. In addition, *n* = 7 studies obtained results that specifically concerned dog ownership [19–21, 38, 48, 49, 52]. While *n* = 4 of these *n* = 6 studies looked at present dog ownership [21, 38, 48, 52], *n* = 1 investigated the longitudinal effects of acquiring a dog [49], and *n* = 1 article also examined the influence of both having a dog now and having had a dog in the past [20]. Eventually, *n* = 5 studies examined present cat ownership [21, 38, 43, 48, 52].

All studies except *n* = 1 [7] examined the association between pet ownership and loneliness. Hereby, *n* = 9 studies employed versions of the UCLA Loneliness Scale [17, 38, 39, 43, 48–50, 52, 53], with *n* = 1 of them also using an additional four-point scale to rate feelings of loneliness during the past week [49]. Moreover, *n* = 1 study relied on such a four-point scale alone [19]. Furthermore, *n* = 1 study employed a composite measure consisted of the UCLA Loneliness Scale and the Health and Retirement Study Psychosocial and Lifestyle Questionnaire [44]. *n* = 1 study relied on the De Jong Gierveld Loneliness Scale [21]. Eventually, *n* = 1 article regarded whether there were any felts of loneliness during the last 2 weeks [18] and *n* = 1 study whether one had the expectation that dog ownership would result in a decrease of loneliness [20]. Taken together, *n* = 2 studies examined the relationship between pet ownership and social isolation [7, 21]. Hereby, *n* = 1 study employed the scale from Bude and Lantermann [32] [21], while the other one quantified social isolation as having contact with others less than once a week.

With respect to the association between owning any kind of pet and loneliness, *n* = 4 out of *n* = 6 studies did not detect a significant relationship between the two variables [17, 18, 44, 48, 50]. However, *n* = 1 study revealed that pet ownership was related to increased levels of loneliness [39]. Concerning the interaction term, having a pet was not found to have a significant effect on loneliness following a social loss in *n* = 1 study [44]. Eventually, *n* = 1 study stated that current or past pet ownership was related to decreased chances of social isolation [7]. As for dog ownership, *n* = 1 study stated that it was negatively associated with loneliness [21], while another study did not explore a significant relationship [48]. In addition, *n* = 1 study that looked at the effect of dog ownership using a longitudinal design found acquiring a dog to be related to decreased levels of loneliness on a single item, which regarded the past week, but not on the UCLA Loneliness Scale [49]. Furthermore, current dog ownership benefitted the opinion that a dog would decrease loneliness, as reported by the participants of *n* = 1 study [20]. Eventually, *n* = 1 study revealed that dog ownership was related to decreased odds of social isolation [21]. Concerning cat ownership, neither the *n* = 2 studies that looked at its association with loneliness [43, 48] nor the *n* = 1 study that examined its relationship to social isolation [21] explored a significant association. Finally, *n* = 3 studies directly compared between owning any kind of pet, owning a dog, and owning a cat with regard to their effect on loneliness [38, 48, 52]. However, none of them found any differences between those groups. Detailed results are presented in Table 3.

**Table 3:** Key findings

First author	Country	Assessment of pet ownership	Assessment of loneliness or social isolation	Study type	Sample characteristics				Results
					Sample description	Sample size	Age	Females in total sample (%)	
Adult population prior to the pandemic									
Antonacopoulos (2010) [50]	Canada	Pet ownership (dichotomous)	UCLA Loneliness Scale (Version 3) (20 items)	Cross-sectional	Individuals who are living alone	*n* = 132	M: 39.4 SD: 14.4 18–78	73.3	According to hierarchical regression, there was no significant association between pet ownership (ref.: non-pet ownership) and loneliness However, pet ownership × social support was associated with decreased levels of loneliness (*ß* = – 0.32, *p* < 0.05)
Antonacopoulos (2017) [49]	Canada	Having acquired a dog (dichotomous)	Feelings of loneliness during the last week, rated on a four-point scale UCLA Loneliness Scale (Version 3) (20 items)	Longitudinal (two waves during 8 months)	Individuals who are living in a town and do not have a dog at the baseline	*n* = 139	M: 36.8 SD: 14.4 18–68	64.0	Regarding the UCLA Loneliness Scale, acquiring a dog (ref.: not acquiring a dog) was not related to diverging levels of loneliness With respect to the single item, ANOVA revealed that acquiring a dog (ref.: not acquiring a dog) was associated with decreased levels of loneliness (*p* < 0.05)
Bennett (2015) [38]	Australia	Pet ownership (dichotomous) Dog ownership (dichotomous) Cat ownership (dichotomous)	UCLA Loneliness Scale-Revised (20 items)	Cross-sectional	Community-dwelling individuals	*n* = 68	M: 71.6 SD: 5.6 65–80	72.1	*T* tests revealed no significant differences between pet owners and non-pet owners, dog owners and non-dog owners, and cat owners and non-cat owners
Branson (2019) [43]	United States	Cat ownership (dichotomous)	UCLA Loneliness Scale Revised (20 items)	Cross-sectional	Community-dwelling individuals without a dog	*n* = 96	M: 76.6 SD: 9.5 60–100	74.0	Logistic regression did not detect loneliness as a significant covariate of cat ownership (ref.: non-cat ownership)
Carr (2020) [44]	United States	Pet ownership (dichotomous) × social loss (dichotomous)	Composite measure (UCLA Loneliness Scale, Health and Retirement Study Psychosocial and Lifestyle Questionnaire) (three items)	Longitudinal (three waves during 8 years)	Health and Retirement Study	*n* = 437	M: 65.6 SD: 10.1 37–88	56	Pet ownership (ref.: non-pet ownership) did not significantly affect changes in loneliness following a social loss
Enmarker (2015) [19]	Norway	Pet ownership (dichotomous)	Loneliness: four-point scale	Cross-sectional	Nord-Trøndelag Health Study	*n* = 12,093	M: 74.8 SD: 6.5 65–101	54.3	“There was a slight difference in pet ownership in relation to loneliness: 16.5% of participants who indicated that they were lonely owned a pet compared with 18% of participants who indicated that they were not lonely.”
Gulick (2012) [52]	United States	Dog ownership (dichotomous) Cat ownership (dichotomous)	UCLA Loneliness Scale (20 items)	Cross-sectional	Individuals who own a dog or cat, utilize services for older people and can communicate in English	*n* = 159	55–72: 50.9% 73–84: 49.1%	100.0	There were no significant differences among loneliness between cat and dog owners
Hajek (2020) [21]	Germany	Dog ownership (dichotomous) cat ownership (dichotomous)	Loneliness: De Jong Gierveld Loneliness Scale (11 items) Social isolation: scale from Bude & Lantermann, 2006 (four items)	Cross-sectional	German Ageing Survey	*n* = 1,160	M: 75.1 SD: 6.4 65–95	65.4	Linear regression showed that dog ownership (ref.: not owning a pet) was related to decreased levels of social isolation (*ß* = – 0.16, *p* < 0.05) and loneliness (*ß* = – 0.12, *p* < 0.1). Cat ownership (ref.: not owning a pet) remained insignificant
McConnell (2011) [53]	United States	Pet ownership (dichotomous)	UCLA Loneliness Scale (20 items)	Cross-sectional	Community sample	*n* = 217	M: 31 SD not specified Range not specified	79	Regarding *t* tests, pet owners (ref.: non-pet ownership) had lower loneliness scores (*p* < 0.08)
Pikhartova (2014) [39]	United Kingdom	Pet ownership (dichotomous)	Revised UCLA Loneliness Scale (three items)	Longitudinal (five waves during 9 years)	English Longitudinal Study of Ageing	*n* = 5,210	M: 61.4 SD not specified Range not specified	55.8	According to logistic regression, pet ownership (ref.: non-pet ownership) was associated with increased odds of loneliness in the cross-sectional analysis (OR: 1.24, 95% CI: 1.06–1.47) In the longitudinal analysis, pet ownership (ref.: non-pet ownership) was also related to higher chances of loneliness (e.g., wave 0 to wave 5: OR: 1.31, 95% CI: 1.03–1.68)
Powell (2018) [20]	Australia	Dog ownership (current or past or not)	Expectation that dog ownership would result in a decrease among loneliness	Cross-sectional	Potential dog owners	*n* = 3,465	18–44: 52.0% 45–64: 39.0% ≥ 65: 9.0%	85.0	According to logistic regression, current dog ownership (ref.: never owned a dog) was significantly related to higher expectations that a dog benefits to a decrease in loneliness (OR: 1.61, 95% CI: 1.19–2.20). Past dog ownership remained insignificant
Rijken (2011) [48]	Netherlands	Pet ownership (dichotomous) Dog ownership (dichotomous) Cat ownership (dichotomous) Dog and cat ownership (dichotomous) Other pet ownership (no cats or dogs) (dichotomous)	UCLA Loneliness Scale Revised (six items)	Cross-sectional	National Panel of People with Chronic Illness or Disability	*n* = 1,410	M: 74.6 SD: 6.4 Range not specified	60.0	With respect to ANOVA, there were no significant differences between the different types of pet ownership among loneliness
Stanley (2014) [18]	United States	Pet ownership (dichotomous)	Felts of loneliness during the last 2 weeks (dichotomized)	Cross-sectional	Primary care patients	*n* = 830	M: 72.2 SD: 8.3 Range not specified	57.8	According to logistic regression, pet ownership (ref.: non-pet ownership) was not significantly associated with loneliness Though, living alone x pet ownership was significantly related to decreased odds of loneliness (OR: 0.20, 95% CI: 0.08–0.50)
Taniguchi (2018) [7]	Japan	Pet ownership (current or past vs. not)	Social isolation: having contact with others less than once a week	Cross-sectional	Ota Genki Senior Project	*n* = 11,233	65–74: 47.7% 75–84: 52.3%	51.6	Referring to mixed-effects cumulative logistic regression models, social isolation was related to decreased chances of current or past pet ownership (ref.: non-pet ownership) (OR: 0.74, 95% CI: 0.66–0.80)
Zasloff (1994) [17]	United States	Pet ownership (dichotomous)	UCLA Loneliness Scale-Revised (number of items not specified)	Cross-sectional	Single students who do not live with a mate, a significant other, or children under the age of 18	*n* = 148	M: 28.4 SD: 8.3 21–53	100.0	There were no significant differences in loneliness among pet owners and non-pet owners
Children/adolescent prior to the pandemic									
Black (2012) [36]	United States	Pet ownership (dichotomous)	UCLA Loneliness Scale Revised (20 items)	Cross-sectional	Rural adolescents who visit public high schools	*n* = 293	M: 15.8 SD: 1.3 13–19	54.1	An ANOVA showed that individuals with pets (ref.: non-pet ownership) had significantly lower loneliness scores (*p* < .001)
Charmaraman (2020) [51]	United States	Pet ownership (dichotomous) dog ownership (dichotomous)	Social isolation: two items	Cross-sectional	Middle school students	*n* = 700	M: 12.7 SD not specified 11–16	52	Social isolation was negatively associated with dog ownership (*ß* = – 0.23, *p* < 0.05, ref.: pet, but non-dog ownership), but not with pet ownership (ref.: non-pet ownership) in general, according to regression analysis
Mueller (2021) [45]	United States	Pet ownership (dichotomous) Dog ownership (dichotomous)	Three-point scale	Longitudinal (two waves in 10 months)	Adolescents visiting Middle schools	*n* = 1,033	M: 12.69 SD: 1.21	50	Dog ownership (ref.: non-dog pet ownership) was related to decreased levels of loneliness (*ß* = – 0.1, *p* < 0.05), according to regression analysis
Rhoades (2015) [37]	United States	Pet ownership (dichotomous)	UCLA Loneliness Scale (three items)	Cross-sectional	Homeless youth who utilize drop-in centers	*n* = 398	M: 21.3 SD: 2.1 Range not specified	27.4	Regarding Chi-square tests, pet ownership (ref.: non-pet ownership) was associated with decreased levels of loneliness (*p* < .05)
Adult population during the pandemic									
Carr (2021) [22]	United States	Dog ownership (dichotomous) Cat ownership (dichotomous)	Composite measure (UCLA Loneliness Scale, Health and Retirement Study Psychosocial and Lifestyle Questionnaire) (three items)	Longitudinal (two waves during 2 years)	Community-based sample	*n* = 473	M: 69.4 SD: 6.1 60–92	66.0	According to the fully adjusted regression model, neither dog ownership (ref.: non-dog ownership) nor cat ownership (ref.: non-cat ownership) was significantly associated with loneliness
Kogan (2021) [23]	Mostly United States	Dog ownership (dichotomous) Cat ownership (dichotomous)	Loneliness: five-point scale Social isolation: five-point scale	Cross-sectional	Dog or cat owners who participated in an online survey	*n* = 5,061	≤ 39: 30% 40–59: 43% ≥ 60: 27%	89	Most of the pet owners reported that their pet would decrease their loneliness (66%) and their feelings of isolation (64%) Regarding binary regression, cat owners were less likely to feel isolated than dog owners (OR: 0.74, 95% CI: 0.64–0.86). Concerning loneliness, there no significant differences between these groups were revealed
Oliva (2021) [46]	Australia	Dog ownership (dichotomous) Cat ownership (dichotomous)	UCLA Loneliness Scale (three items) Loneliness during COVID-19 lockdown, rated on a four-point scale	Cross-sectional	Individuals living alone	*n* = 384	M: 50.9 SD: 15.1 23–89	85.4	Referring to hierarchical logistic regression, dog ownership (ref.: non-dog ownership) was associated with decreased levels of loneliness among both measures (e.g., UCLA Loneliness Scale: *ß* = – 0.71, *p* < 0.05). Cat ownership (vs. non-cat ownership) remained insignificant
Phillipou (2021) [24]	Australia	Pet ownership (dichotomous)	UCLA Loneliness Scale-Revised (number of items not specified)	Cross-sectional	Covid-19 and you: mentaL heaLth in AusTralia now survEy	*n* = 263	M: 25.1 SD: 14.2 range not specified	84.2	Pet ownership (ref.: non-pet ownership) was not significantly related to loneliness
Ratschen (2020) [47]	United Kingdom	Pet ownership (dichotomous)	UCLA Loneliness Scale (three items)	Cross-sectional	General population	*n* = 5,926	18–24: 7.1% 25–34: 17.5% 35–44: 16.8% 45–54: 23.8% 55–64: 22.2% 65–70: 7.1% ≥ 70: 5.6%	78.6	Looking at linear regression models, pet ownership (ref.: non-pet ownership) was associated with a decreased height of loneliness (*p* < 0.01)
Children/adolescent during the pandemic									
Mueller (2021) [45]	United States	Pet ownership (dichotomous) Dog ownership (dichotomous)	Three-point scale	Longitudinal (two waves in 10 months)	Adolescents visiting Middle schools	*n* = 357	M: 12.69 SD: 1.21	50	Pet ownership (ref.: non-pet ownership) was significantly associated with increased loneliness during COVID-19 (*ß* = 0.12, *p* < 0.05) Pet owners (ref.: non-pet owners) reported significantly higher increases in loneliness during COVID-19 (*ß* = 0.14, *p* < 0.01)

#### Adult population during the pandemic

In sum, *n* = 5 studies [22–24, 46, 47] investigated the association between pet ownership, loneliness, and social isolation regarding the COVID-19 pandemic. Therefore, they relied on data from Australia (*n* = 2) [24, 46], the United States (*n* = 2) [22, 23], and the United Kingdom (*n* = 1) [47]. Only *n* = 2 studies took a look at owning a pet in general [24, 47], while *n* = 3 articles specifically treated cat ownership and dog ownership [22, 23, 46]. The assessment of loneliness vastly differed among the studies: *n* = 3 of them used the UCLA Loneliness Scale [24, 46, 47], with one additionally employing a four-point scale to quantify loneliness during the COVID-19 lockdown [46]. Furthermore, *n* = 1 study relied on a composite measure with items from the UCLA Loneliness Scale and the Health and Retirement Study Psychosocial and Lifestyle Questionnaire [22]. Finally, *n* = 1 study employed a five-point scale to assess loneliness [23]. Eventually, *n* = 1 study investigated the association between pet ownership and social isolation, using a five-point scale to quantify the latter [23].

Among the *n* = 2 studies that looked at the relationship between owning any kind of pet and loneliness, *n* = 1 found pet ownership to be associated with decreased chances of loneliness [47], while the other one did not reveal a significant result [24]. Regarding dog ownership, *n* = 2 studies [23, 46] found it to be related to decreased of levels of loneliness, while *n* = 1 study [22] did not detect a significant effect. In addition, *n* = 1 study showed that dog ownership was related to decreased chances of self-perceived social isolation [23]. Concerning cat ownership, *n* = 1 study reported that it was associated with decreased levels of perceived loneliness and social isolation [23], whereas *n* = 2 studies did not reveal a significant relationship between cat ownership and loneliness [22, 46]. Finally, *n* = 1 study revealed that cat owners were less likely to feel socially isolated, but not less likely of feeling lonely than dog owners [23]. Detailed findings are presented in Table 3.

### Children/adolescent

#### Children/adolescent prior to the pandemic

The relationship between pet ownership, loneliness, and social isolation regarding the situation before the outbreak of COVID-19 was investigated by *n* = 4 studies [36, 37, 45, 51]. All of them relied on data from the United States and investigated whether one owned any kind of a pet during the time the study was conducted. In addition, *n* = 2 studies also examined dog ownership [45, 51]. In sum, *n* = 3 studies examined the relation between pet ownership and loneliness. While *n* = 2 studies were employing the UCLA Loneliness Scale [36, 37], *n* = 1 used a three-point scale [45]. Eventually, *n* = 1 article referred to social isolation, assessing it with two items [51].

Regarding loneliness, the studies pointed towards a positive effect of pet ownership: *n* = 2 studies stated that pet ownership in general was related to decreased levels of loneliness [36, 37], while *n* = 1 did not reveal a significant association [45]. The study that looked at the association between pet ownership in general and social isolation did not reveal a significant relation as well [51]. However, both studies that examined the role of dog ownership found out that it was related to decreased levels of loneliness [45] as well as to decreased levels of social isolation [51]. Details are displayed in Table 3.

#### Children/adolescent during the pandemic

*n* = 1 study investigated the association between pet ownership and loneliness during the COVID-19 pandemic [45]. Hereby, it used data from the United States to regard pet ownership in general as well as dog ownership. Loneliness was assessed using a three-point scale.

The study found out that pet owners reported significantly higher increases in loneliness during COVID-19. Once more, a detailed presentation is provided in Table 3.

## Discussion

The aim of our systematic review was to synthesize the evidence on the association between pet ownership, loneliness, and social isolation. It includes *n* = 24 studies which examine this relationship.

Regarding the findings that do not specifically relate to the COVID-19 pandemic, most of the studies that investigated an adult sample did not detect a significant association between pet ownership and loneliness, with the remaining ones both pointing towards both a positive and a negative relationship. However, regarding social isolation, the one study that investigated its relation to pet ownership in general stated that it may be able to reduce social isolation [7]. This goes hand in hand with the finding of the only other study out of the ones relying on an adult sample that were executed before the outbreak of COVID-19, which also stated that dog ownership may reduce social isolation [21]. Though, it is impossible to formulate a general rule based on the findings of only two articles. Eventually, cat ownership was not found to be related to loneliness or social isolation by any of the studies in this subsample.

Regarding the evidence that was obtained on adults since the outbreak of COVID-19, half of the few studies stated that dogs may assist in reducing loneliness or social isolation. Notwithstanding that, ownership of any kind of pet as well as cat ownership did not seem to have a significant influence on loneliness and social isolation. However, one study that directly compared dog and cat owners stated that the latter ones were less likely to perceive themselves as socially isolated [23]. Due to the low number of studies in this subsample, it does not seem clear whether this is just an exception to the tendency of the three other studies.

The number of studies examining child and adolescent samples is not high enough to draw general conclusions as well. However, the studies that were conducted before the outbreak of COVID-19 may suggest a negative association between pet ownership in general as well as dog ownership and loneliness. Though, when it comes to the study that investigated the relationship after the outbreak of COVID-19, owning any kind of pet was found to be related to higher levels of loneliness, and dog ownership was shown to have a tendency to increase the changes in loneliness compared to the pre-pandemic level [45].

Finally, there do not seem to be major differences between dogs, cats, or other companion animals, as most of the studies did not detect any significant discrepancies between dog owners, cat owners, and owners of another kind of pet among their loneliness and social isolation levels. Though dog ownership is more often associated with significantly better outcomes regarding loneliness and social isolation than cat ownership among the small part of the studies that did explore any significant results, most of the direct comparisons between dog owners and cat owners did not reveal any significant differences. Considering findings from previous studies, this may seem somewhat surprising, as dogs are assumed to be particularly beneficial in terms of getting to know new people, as they have to be walked out every day [21]. However, other studies do only report differences between different pet types that concern the quality, but not the quantity of social relationships (as outcome measure) [9]. Such a neglect of the quantitative aspects of social relationships could partly explain why most of the studies did not reveal any significant differences between owners of different pet types, as both loneliness and social isolation can also refer to them, though especially loneliness also holds the qualitative features of one’s relationships in high esteem. Eventually, some of the variety in the findings may also be explained by the fact that most of the studies did not account for the number of pets owned or the health status of the pets. This could be seen as a limitation when the effort that particularly different dog races are causing is taken into account.

Concerning the general insignificance of pet ownership when it comes to loneliness among adult populations, past research already stated that this association can vary due to differences in the samples which are investigated. For instance, the results by Hajek and König suggested that dog ownership is related to reduced levels of loneliness and social isolation particularly among women, compared to not owning a pet [21]. The authors assumed that this is due to a higher willingness of women to substitute contacts with human beings through contacts with their dogs in case of a reduced social network. On the other hand, Stanley et al. revealed that gender does not moderate the relationship between pet ownership and loneliness [18]. Finally, the two studies that solely focused on female samples did not detect any significant differences between pet owners and non-pet owners [17, 52]. Therefore, it does not seem to be possible to provide a clear statement about any sex differences among the pet effect in terms of loneliness or social isolation. Meanwhile, the finding of Pikhartova et al. was that pet ownership is related to higher odds of loneliness [39]. However, they hypothesized that this may be a case of reverse causality, as pet ownership could also be a response to loneliness. This means that it may not increase but protect from loneliness regarding individuals with a tightly limited social network. Regarding these studies, it seems probable that there is a significant relationship between pet ownership and loneliness among specific subgroups. Though, all studies included in this review taken together, it seems difficult to draw any conclusions in terms of the applicability of the Attachment Theory among general populations, as the overall evidence remains mixed.

Regarding children and adolescents, the results of the few studies which investigated the relation between pet ownership, loneliness, and social isolation among such samples indicated a higher possibility of any benefits of pet ownership concerning the two outcome variables. Thus, in this case, the Attachment Theory is more likely to hold. Nevertheless, some studies also suggested that attachment to a pet is associated with a higher importance of non-social activities, such as gaming, in children’s life [51]. Therefore, it also seems possible that pet ownership is more likely among children who tend to be more alone (reverse causality).

Finally, COVID-19 may have caused some major differences in the association between pet ownership, loneliness, and social isolation both among adults and among the youth. While studies that were conducted since the pandemic began are more likely to state that pet ownership is related to decreased chances of loneliness among adults, the study that examined the same association among adolescents suggests the opposite: Pet ownership was related to a higher increase of loneliness during the pandemic [45]. The authors once more suggested that this may be explained by selection effects with regard to the individuals who choose to own pets. Meanwhile, studies which revealed the positive effects of pet ownership during the pandemic stressed the social aspect related to holding companion animals, especially dogs. As they must be walked out every day, they could bring one in touch with one’s social environment [22]. This may be particularly important during a lockdown, when the quantity of social contacts may be reduced, and could assist in explaining the higher importance of pet ownership when it comes to loneliness, compared to the years before COVID-19 arose.

Our summary of the existing articles which regard to the relationship between pet ownership, loneliness, and social isolation may provide some proposals for further research on that topic. First, it seems promising to not only investigate loneliness, but also social isolation. Although both constructs are related to each other, social isolation may be associated with pet ownership, while loneliness is less likely to be. Therefore, a more differentiated perspective on constructs that represent (unmet) social needs could provide new insights. Second, all the studies included in our review were conducted in high-income countries, with the majority of them relying on samples from English-speaking countries. Meanwhile, studies examining the association between pet ownership, loneliness, and social isolation among low- and middle-income countries are completely missing. Third, there is also a lack of both longitudinal studies and studies that examine child or adolescent samples. As the latter individuals were more often found to benefit from pets in terms of loneliness, additional investigations could clarify whether this first impression can take hold in other contexts as well. Furthermore, longitudinal studies could resolve the uncertainties when it comes to causality. Especially the influence of loneliness and social isolation on the odds of pet ownership could also be examined.

As for the strengths of our review, it is, to the best of our knowledge, the first systematic review which synthesizes the evidence on the association between pet ownership, loneliness, and social isolation. As studies that solely focused on specific samples were excluded, its results pertain to larger population groups. Study screening, data extraction, and quality assessment were executed by two reviewers to prevent bias and ensure a sufficient quality of our report.

There are also two limitations of our current systematic review that should be mentioned. First, a meta-analysis was not carried out due to the heterogeneity between the studies, relying on recommendations that alert to biased estimates due to those differences [54]. Second, there was only limited potential to draw general conclusions regarding certain questions, e.g., the association between pet ownership and social isolation, due to an insufficient number of studies.

## Conclusion

All in all, the findings of the studies included in our review did not point towards a significant association between pet ownership on loneliness, but possibly between pet ownership and social isolation among adult populations. However, COVID-19 may have led to a more pronounced association between pet ownership and loneliness. Regarding children and adolescents, pet ownership may reduce loneliness in general, but there could be some changes to this relationship due to the COVID-19 pandemic. It is worth mentioning that all studies included in our review were conducted in high-income countries. Future research may also look at the effect of pet ownership in low- and middle-income countries, especially regarding probably more instrumental roles obtained by animals in these locations. In addition, there is a lack of longitudinal studies and of studies investigating social isolation. Thus, future longitudinal studies examining the association between pet ownership and social isolation are desirable.
